# Radio-Activity of Mineral Waters

**Published:** 1912-02

**Authors:** 


Radio-Activity of Mineral Waters. G. A. Persson (sic) of Mt. Clemens, *Lancet-Clinic,* October 14, 1911, states that radio-activity has been demonstrated in the Carlsbad, Pöstyen, Arkansas Hot Springs, Yellow Stone Park and other waters. (We believe, also in the waters of Bath, England, which has been a resort since the Roman occupancy, at least, and where, indeed, one can still see the very much restored Roman bath house.) Persson reproduces radiographs to show a high degree of radio-activity in the waters of Mt. Clemens.

In the Carlsbad waters, radio-activity has been found to be higher in the cooler springs, and has even been demonstrated in the gases. Much of the scepticism regarding the beneficial effects of sojourns at springs has been dissipated by the demonstration of radium and its congeners and it is altogether likely that actinic emanations explain the superior results of visits to springs, as compared with the home use of stale water, supplanting the former explanation of the benefits of rest, change, and suggestion. This explanation, is, however, a double-bladed sword for it is perfectly possible that radio-therapy may be obtained at home nor must we forget that any therapeutic agent requires careful dosage and study of contraindications.

